# Pantothenate Kinase 1 Inhibits the Progression of Hepatocellular Carcinoma by Negatively Regulating Wnt/β-catenin Signaling

**DOI:** 10.7150/ijbs.67842

**Published:** 2022-01-24

**Authors:** Yuyuan Zi, Jie Gao, Chenglv Wang, Yidi Guan, Linzhao Li, Xinxin Ren, Lan Zhu, Yun Mu, Shuang-hui Chen, Zimei Zeng, Zhen Cao, Zhuoxian Rong, Pan Chen, Xiuping Zhang, Tao Chen, Haiguang Xin, Xuebing Li, Zhi Li, Lunquan Sun, Yuezhen Deng, Nan Li, Yingjie Nie

**Affiliations:** 1 Department of Oncology, Xiangya Cancer Center, Xiangya Hospital, Central South University, Changsha 410008, China.; 2 NHC Key Laboratory of Pulmonary Immune-related Diseases, Guizhou Provincial People's Hospital, Guiyang 550002, China.; 3 Hunan Cancer Hospital, The Affiliated Cancer Hospital of Xiangya School of Medicine, Central South University, Changsha, 410013, China.; 4 Faculty of Hepato-Biliary-Pancreatic Surgery, The First Medical Center of Chinese People's Liberation Army (PLA), General Hospital, Beijing, China.; 5 State Key Laboratory of Respiratory Disease, National Clinical Research Center for Respiratory Disease, Guangzhou Institute of Respiratory Health, the First Affiliated Hospital of Guangzhou Medical University, Guangzhou 510120, China.; 6 Department of Infectious Disease, Ruijin Hospital, Shanghai Jiao Tong University School of Medicine, Shanghai 200025, China.; 7 Tianjin Key Laboratory of Lung Cancer Metastasis and Tumor Microenvironment, Tianjin Lung Cancer Institute, Tianjin Medical University General Hospital, Tianjin, P. R. China.; 8 Key Laboratory of Molecular Radiation Oncology Hunan Province, Changsha 410008, China.; 9 National Clinical Research Center for Geriatric Disorders, Department of Geriatrics, Xiangya Hospital, Central South University, Changsha 410008, China; 10 Hunan International Science and Technology Collaboration Base of Precision Medicine for Cancer, Changsha 410008, China.; 11 Center for Molecular Imaging of Central South University, Xiangya Hospital, Changsha 410008, China.; 12 Department of Hepatic Surgery I (Ward I), Shanghai Eastern Hepatobiliary Surgery Hospital, Shanghai 200438, China.

**Keywords:** hepatocellular carcinoma, Wnt/β-catenin signaling, PANK1, protein kinase activity, CK1α

## Abstract

Hyperactivation of Wnt/β-catenin signaling has been reported in hepatocellular carcinoma (HCC). However, the mechanisms underlying the hyperactivation of Wnt/β-catenin signaling are incompletely understood. In this study, Pantothenate kinase 1 (PANK1) is shown to be a negative regulator of Wnt/β-catenin signaling. Downregulation of PANK1 in HCC correlates with clinical features. Knockdown of PANK1 promotes the proliferation, growth and invasion of HCC cells, while overexpression of PANK1 inhibits the proliferation, growth, invasion and tumorigenicity of HCC cells. Mechanistically, PANK1 binds to CK1α, exerts protein kinase activity and cooperates with CK1α to phosphorylate N-terminal serine and threonine residues in β-catenin both in vitro and in vivo. Additionally, the expression levels of PANK1 and β-catenin can be used to predict the prognosis of HCC. Collectively, the results of this study highlight the crucial roles of PANK1 protein kinase activity in inhibiting Wnt/β-catenin signaling, suggesting that PANK1 is a potential therapeutic target for HCC.

## Introduction

Hepatocellular carcinoma (HCC) is one of the most common malignancies 1. Its occurrence is related to environmental factors 2, dietary factors and hereditary changes 3. Liver cirrhosis, hepatitis virus infection, and aflatoxin exposure are risk factors for liver cancer 4-6. The occurrence and progression of liver cancer are characterized by significant familial aggregation 7, suggesting that hereditary changes play an important role.

The Wnt/β-catenin signaling pathway is abnormally activated in HCC 8. β-catenin is the core molecule in this signaling pathway. In the Wnt-OFF state, the β-catenin destruction complex in the cytoplasm controls β-catenin stability. The β-catenin destruction complex is composed of Axin, APC, CK1α and GSK3β, and is responsible for phosphorylating the N-terminal serine and threonine residues of β-catenin 9. Phosphorylated β-catenin is ubiquitinated after being recognized by β-TrCP, and is then degraded via the proteasome pathway. After the Wnt ligand binds to the cell membrane receptors LRP5/6 and Frizzled, a conformational change in the C-terminus of Frizzled takes place, triggering the recruitment of DVL. Then, DVL recruits Axin through their DIX domain, thereby causing disintegration of the β-catenin destruction complex. As a result, β-catenin accumulates in the cytoplasm and is then translocated into the nucleus, activating the transcription of downstream genes (e.g., Axin2, c-Myc, and CCND1) 10. Activation of the Wnt/β-catenin signaling pathway promotes the proliferation, invasion, and metastasis of HCC cells 11, 12. In addition, the Wnt/β-catenin signaling pathway promotes glutamine synthesis and activates the mTOR signaling pathway in HCC 13.

Approximately 20-30% of HCC patients harbor constitutively activating mutations in β-catenin, and Axin was deleted in approximately 15-20% of patients 14. However, Axin and β-catenin mutations are insufficient to explain why the Wnt/β-catenin signaling pathway is abnormally activated in 70% of HCC patients 15. Therefore, other mechanisms underlying the activation of the Wnt/β-catenin signaling pathway in HCC exist. To reveal the mechanisms by which the Wnt/β-catenin signaling pathway is activated and identify new targets in this pathway, a siRNA library targeting 720 human kinases was screened 16, and the results suggested that pantothenate kinase (PANK1) is one of the potential negative regulator in the Wnt/β-catenin signaling pathway.

The PANK family has four members: PANK1, PANK2, PANK3 and PANK4 17. PANK1, which is the rate-limiting enzyme in de novo synthesis of coenzyme A (CoA), is responsible for phosphorylating pantothenic acid. PANK1 controls the synthesis of CoA, thereby controlling the ratio of CoA to acetyl-CoA and exerting an important effect on global metabolism 18. Systemic knockout of PANK1 followed by knockout of PANK2 in neurons was found to result in a decrease in the content of short-chain acyl-CoA 19. In Leptin knockout mice, hyperglycemia and hyperinsulinemia can be alleviated by further knockout of PANK1 expression 18. PANK1, whose expression is induced by P53 and PPARα, is an important molecule mediating the metabolic regulatory function of P53 20. Bezafibrate, an agonist of PPARα, induces the expression of PANK1 21. The enzymatic activity of PANK1 can be inhibited by acetyl-CoA, malonyl-CoA, and long chain acetyl-CoA 22. At present, there are very few reports on the role of PANK1 in tumors. Acute myeloid leukemia patients with high expression of pantothenate kinase have longer survival times 17.

In the present study, the expression pattern and functions of PANK1 in HCC were investigated, and the regulation of the Wnt/β-catenin signaling pathway by PANK1 was studied.

## Materials and Methods

### Cell culture and tissue samples

HCC cell lines (Huh7, QGY-7701, 7404, MHCC97, and HepG2), normal liver cells (HHL-5) and 293T were all obtained from the Cell Bank of the Chinese Academy of Sciences. The PVTT cells were established as described previously 23. The QGY-7701 and 7404 HCC cells were cultured in 1640 medium, while the others were cultured in DMEM. 10% fetal bovine serum (FBS) and antibiotics (100 U/mL of penicillin and 100 µg/ml of streptomycin) were added to all media. All cells were cultured in an incubator (5% CO_2_, 37°C). Cell transfection was performed using Lipofectamine 8000 according to the manual. The Wnt3a conditional medium was prepared from the L-Wnt3a cells according to the method from the ATCC.

The HCC tissues and paired adjacent tissues came from the Shanghai Eastern Hepatobiliary Surgery Hospital, Second Military Medical University. All tissue samples were collected with the informed consent of the patients, and were used to study the expression level of PANK1. The clinical features of 72 HCC patients were listed in Table S1. This study was approved by the Ethics Committee of the Second Military Medical University.

### Plasmid and transfection

The CDS sequence of PANK1 was inserted into the pLVX-IRES-puro vector. The shRNA targeting PANK1 was designed with reference to the information on the Sigma website and cloned into the pLKO.1-puro vector. The shRNA sequence was listed in Table S2. The lentivirus was packaged in 293T cells with the packaging vectors psPAX2 and pMD2.G. After being concentrated at PEG8000, the virus solution was centrifuged (1600g, 4°C) for 1h. The supernatant was removed, and dissolved in 2ml DMEM. The cells were seeded into a 6-well plate at a density of 50%-60%. The next day, 400µl of lentivirus was added to and incubated with the cells in a constant temperature incubator overnight. Two days after infection with the lentivirus, the cells were cultured with puromycin (1 µg/mL) for 4 days. Then, stable cell lines were pooled. The expression of PANK1 was detected by western blot.

### qPCR

TRIzol (Invitrogen) was used to extract RNA, and then 1μg of RNA was reversely transcribed into cDNA using the PrimeScript™ RT kit (Takara) according to the instructions. The SYBR Green kit and CFX96 real-time fluorescent quantitative PCR detection system (Bio-Rad, Richmond, CA, USA) were used for qPCR. Taking 18S as internal reference, the Ct (18S-PANK1) method was used to calculate the expression of the target gene. See Supplementary Table 2 for details on the primer sequence.

### Western blotting

The cells were cleaned twice with PBS, and lysed on ice in the RIPA Lysis buffer, which contained a protease inhibitor and a phosphatase inhibitor. The supernatant was collected after the cell lysis was centrifuged. Then the protein concentration was quantified using the BCA protein detection kit. Equal amounts of protein were taken for SDS-PAGE. After separation, the protein was transferred onto the PVDF membrane and incubated with a primary antibody at 4°C overnight. Then, they were incubated with the HRP-conjugated secondary antibody for 1-2h. The immune signal was detected with a chemiluminescence reagent (Millipore, WBKLS0050), and analyzed with Image Lab software. The primary antibodies used in this experiment were as follows: PANK1 (Proteintech, 11768-1-AP, 1:1000), tubulin (Santa Cruz Biotechnolog, sc-5286, 1:4000), GSK3β (BD Transduction Laboratories™, 610202, 1:1000), Flag (Sigma, F9291; 1:3000), Myc (Santa Cruz, sc-40, 1:2000), β-catenin (BD Transduction Laboratories™, 610154, 1:1000), Phospho-β-Catenin (Cell Signaling Technology, 9561S, 1:1000), and Ubiquitin (Proteintech, 10201-2-AP, 1:1000), CK1α (Proteintech, 55192-1-AP, 1:1000).

### Immunohistochemistry (IHC)

The tissue arrays were obtained from the Shanghai Eastern Hepatobiliary Surgery Hospital, Second Military Medical University. The clinical features of the tissue array were listed in Table S3. After dewaxing and rehydration, the tissue sections were put in the EDTA solution, and antigen recovery was performed at 100°C for 30min. After natural cooling to room temperature, the activity of endogenous peroxidase was blocked. After being washed twice in PBS, the tissue sections were incubated with the PANK1 antibody (Sigma, SAB2700892, 1:100) and β-catenin antibody (BD Transduction Laboratories™, 610154, 1:100) at 4°C overnight. The next day, the sections were washed twice in PBS and incubated with the secondary antibody at room temperature for 1h. The immunohistochemical signal was detected with 3,3,0-diaminobenzidine (DAB), and hematoxylin was used for nuclear staining of all the sections. Both the staining intensity and protein expression level were automatically scored by the Vectra 2 system. The PANK1 scores less than 98.2 were identified as “low expression of PANK1”; PANK1 scores greater than or equal to 98.2 were identified as “high expression of PANK1”; β-catenin scores less than 27.8 were identified as “low expression of β-catenin”; and β-catenin scores greater than or equal to 27.8 were identified as “high expression of β-catenin”. The survival curve was drawn using the Kaplan-Meier method, and the log-rank test was used for survival analysis.

### Immunoprecipitation

In order to measure the interaction between exogenous PANK1 and CK1α, the tagged PANK1 and CK1α plasmids were transferred into 293T cells. 48h after transfection, the cells were lysed with an IP lysis buffer (50mM Tris-HCl, pH 8.0, 150mM NaCl, 1%NP-40, protease and phosphatase inhibitor). The supernatant was collected after centrifugation. Flag-beads (Sigma, A2220) or Myc-beads (Bimake, B62301) were added to the supernatant for incubation overnight at 4°C. The next day, the beads were washed 3 times in wash buffer (50mM Tris-HCl (pH 8.0), 150mM NaCl, 1% NP-40), with 1×loading buffer added, and heated at 100°C for 5 min. Then, the supernatant was taken for western blot analysis.

To detect whether there was any interaction between the endogenously expressed PANK1 and CK1α in the HCC cells, after the cells were treated, an IP lysis buffer containing protease and phosphatase inhibitor was used for lysis. Equal amounts of protein were taken, and 0.25μg of PANK1 antibody was added for incubation overnight at 4°C. The next day, 40μL of Protein A/G beads (bimake, B23202) was added for incubation overnight at 4°C. The beads were washed 3 times in the wash buffer, then 1×loading buffer was added for western blotting analysis.

### Luciferase reporter assay

Cells with 50% confluence were seeded into a 12-well plate. Then, the cells underwent co-transfection with 0.1μg of expression vector, 0.05μg of reporter plasmid, and 0.02μg of Renilla luciferase. 24h after the transfection, the cells were incubated with lithium chloride (LiCl) or Wnt3a for 8h. Then, the dual-luciferase reporter assay system (Beyotime, RG088M) was used to test the reporter gene activity. The experiment was repeated three times.

### In vitro kinase reaction assay

Flag-PANK1 and HA-GSK3β were transfected into PVTT cells. 48h later, Flag-PANK1 and HA-GSK3β, were purified by CO-IP, and competitively eluted with Flag-tag polypeptide, obtaining Flag-PANK1 and HA-GSK3β protein. IPTG was used to induce the expression of GST-β-catenin (N-terminus) and GST-CK1α. Briefly, the E. Coli were collected, and the IP lysis buffer was used for resuspension and lysis. Then, they were sonicated at 20% power for 9s, and treated in an ice bath for 1min. The operation was repeated 3 times. Then, the solution was centrifuged (4°C, 14000rpm) for 10min, and the supernatant was collected. 20μL of GST beads (GE Healthcare, 17-0756-01) was added and incubated with the supernatant at 4°C for 2 hours. The beads were then washed 3 times in the wash buffer. 10 mM ATP (Cell Signaling Technology, 9804S), 10×kinase buffers (Cell Signaling Technology, 9802S), Flag-PANK1 and HA-GSK3β were added, followed by the reaction at 32°C for 45 min. At the end of the reaction, the loading buffer was added, and the western blot analysis was performed.

### Ubiquitination analysis

The stable cell line was treated with MG132 at a final concentration of 20μM for 10 hours. The cells were lysed in an IP lysis buffer, the supernatant was collected and then 0.5μg of β-catenin antibody was added for incubation overnight at 4°C. The next day, 40μL of Protein A/G beads (bimake, B23202) was added and incubated with the supernatant for 4 hours at 4°C. The beads were washed 3 times in the wash buffer, and then 1×loading buffer was added for 5min of heating at 100°C. The supernatant was taken for western blot analysis.

### Tumorigenesis in nude mice

All work related to animals was approved by the Animal Ethics Committee of the Central South University. Nude mice aged 4-6 weeks were used, with 4 in each group. 4×10^6^ control cells and Huh7 cells with overexpressed PANK1 were injected subcutaneously at each point. The tumor volume was calculated according to the following formula: volume = (length×width^2^)/2. The mice were killed in the 7th week after the start of the experiment to harvest the tumors.

### Soft agar

When the confluence reached 60-70%, the cells were digested, and a cell suspension was prepared. Lower-layer gel (20% FBS, 40% 2×RPMI1640 (Basal Medium Eagle) 40% 1.25% Agar) was prepared. 400μL of gel was added to each well in the 24-well plate. The gel was placed in an incubator at 37°C. The gel was solidified for later use. Upper-layer gel (25% FBS, 37.5% 2×RPMI1640, 37.5% 1% Agar, 0.8% 2mM L-glutamine) was prepared and mixed evenly with the cell suspension. 400μl (containing 4×10^3^ cells) was added to each well and placed in a constant temperature incubator (37°C, 5% CO_2_) for 10-14d. 5 fields of view were selected randomly under the microscope for colony counting.

### CCK8

The cells were seeded into a 96-well plate, with 1×10^3^ cells in each well, and cultured in the incubator (37°C, 5% CO_2_). The next day, the old medium was replaced with a fresh medium containing 10% CCK8, which was placed in the incubator for 2h of incubation to measure its absorbance at 450nm. Tests were performed on days 1, 3, 5 and 7.

### EdU

The cells were seeded into a 96-well plate, with 1×10^4^ cells in each well. The cells were measured using the Cell-Light EdU Apollo567 In Vitro Kit (RiboBio, C10310-1), while a fluorescent microscope was used to take photographs for analysis.

## Results

### PANK1 is downregulated in HCC

**Figure 1:**
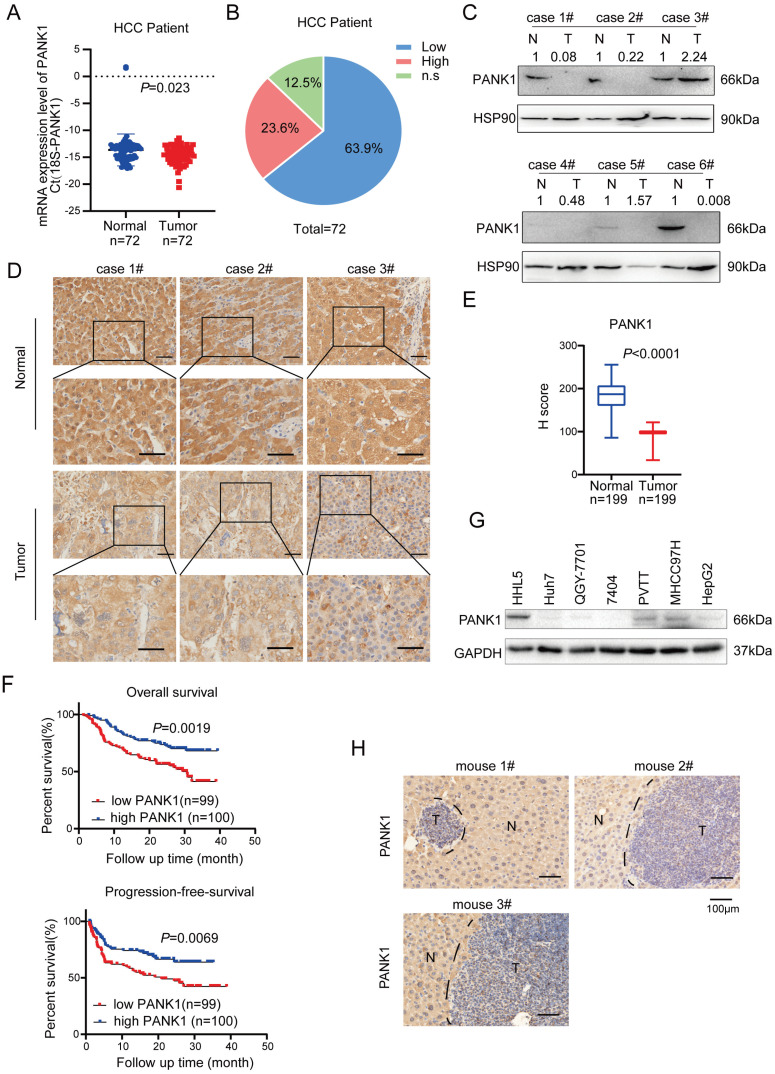
** PANK1 is down-regulated in HCC. (A)** qPCR was used to measure the mRNA level of PANK1 in 72 cases of HCC tissues and adjacent tissues. The diagram took Ct (18S-PANK1) as the ordinate. 18S was used for internal reference. **(B)** This pie chart showed the changes of PANK1 mRNA in HCC tissues and adjacent tissues from the same patient. “Low”, PANK1 expression was lower in tumor tissues than in adjacent tissues; “High”, PANK1 expression was higher in tumor tissues than in adjacent tissues. N.S, no change. **(C)** Western blot was used to measure the level of PANK1 protein in 6 cases of HCC tissues and paired adjacent tissues. N, the adjacent tissue; T, the tumor tissue. **(D)** Immunohistochemistry (IHC) was used to measure the level of PANK1 protein in 3 cases of HCC tissues (tumor) and adjacent tissues (normal). **(E)** The level of PANK1 protein in the adjacent tissues and HCC tissues in the HCC tissue array was scored. After immunohistochemical staining, the tissue array (including 199 cases of HCC tissues and paired normal tissues) was scored. **(F)** Survival analysis. Analysis of the correlation between expression of PANK1 and overall survival/progression-free survival. **(G)** Western blot was used to measure the level of PANK1 protein in HCC cell lines (HHL5, Huh7, QGY-7701, 7404, MHCC97H, PVTT, and HepG2). **(H)** IHC was used to measure the level of PANK1 protein in the DEN-induced HCC model mice. N, the normal tissues; T, the tumor tissues. The scale bars were indicated.

In previous study 16, we have screened the kinases targeting WNT/β-catenin pathway using the siRNA library, and we got 181 kinases with fold change above 2.0. By co-analysis with TCGA database, 47 differentially expressed kinases were obtained in HCC. Further KEGG enrichment analysis revealed that 8 out of 47 kinases were related to metabolism. Of these 8 kinases, GALK1, PCK1 and PANK1 were highly expressed in normal liver tissues and down-regulated in hepatocellular carcinoma (HCC). Considering that the functions for PANK1 in the tumorigenesis were poorly understood, we chose to study the roles of PANK1 in the regulation of WNT/β-catenin pathway in HCC (Figure S1A). To study the expression pattern of PANK1 in HCC, first, the mRNA levels of PANK1 in 72 pairs of HCC tissues were measured, revealing the down-regulation of the PANK1 mRNA level in HCC tissues (Figure 1A). Further analysis showed that compared with that in the normal adjacent tissues, the PANK1 mRNA level was reduced in 63.9% of the HCC tissues (Figure 1B). Then, western blotting was used to detect the PANK1 protein level in 6 HCC tissues and paired adjacent tissues. The experimental results showed that the PANK1 protein level was significantly downregulated in HCC tissues (4/6) (Figure 1C). To further analyze the correlations between the PANK1 protein level and the clinical characteristics of HCC, the protein levels of PANK1 in tissue array containing 199 HCC tissues and paired adjacent tissues were measured. Immunohistochemical staining of the array showed that the PANK1 protein level was significantly decreased in the HCC tissues (Figure 1D-E) and that the levels of PANK1 protein were significantly negatively correlated with tumor size (Table 1). Moreover, the PANK1 protein level was significantly positively correlated with overall survival and progression-free survival in HCC patients (Figure 1F), these results were consistent with the data derived from the TCGA database and KM plot database. In the TCGA database, the expression of PANK1 in HCC tissues was lower than that in the adjacent tissues. Meanwhile, PANK1 expression was positively correlated with survival of HCC patients. In addition, the highest PANK1 expression was observed in normal liver tissues according to The Human Protein Atlas database (Figure S1B-D). However, the levels of PANK1 protein were not correlated with the serum bio-marks (Figure S2A-S2M). Furthermore, expression of PANK1 was downregulated in most HCC cell lines (Figure 1G). In addition, in the mouse model of DEN-induced HCC, the expression of PANK1 was downregulated in HCC tissues (Figure 1H). In summary, the expression of PANK1 is downregulated in HCC tissues, suggesting that PANK1 may play an important role in the occurrence and progression of HCC.

### Overexpression of PANK1 inhibits the proliferation, growth, colony formation, and invasion of HCC cells

**Figure 2:**
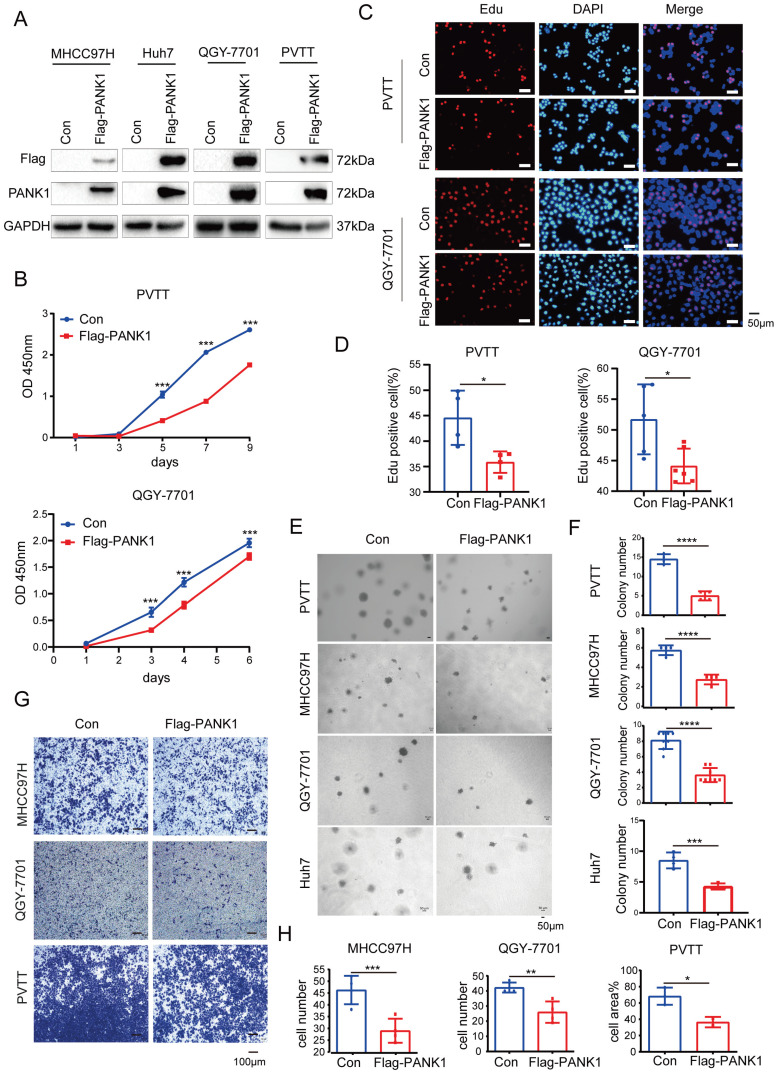
** PANK1 inhibits proliferation, growth, and invasion of HCC cells. (A)** PANK1 was overexpressed in MHCC97H, Huh7, QGY-7701and PVTT cells. MHCC97H, Huh7, QGY-7701 and PVTT cells were infected with a lentivirus with overexpressed Flag-PANK1. After puromycin screening, the expression of Flag-PANK1 in the surviving cells was measured by western blot using anti-Flag or anti-PANK1 antibodies. **(B)** A CCK8 assay was performed to measure the effect of PANK1 overexpression on the growth of PVTT and QGY-7701 cells. **(C-D)** EdU assay was performed to examine the effect of PANK1 expression on the proliferation of PVTT and QGY-7701 cells. The positive cells were quantified and analyzed. **(E-F)** A soft agar colony formation assay was performed to examine the effect of PANK1 expression on PVTT, MHCC97H, QGY-7701 and Huh7 colonies. The colonies were quantified and analyzed. **(G-H)** Invasion assay using the Transwell was conducted to examine the effect of PANK1 expression on the invasion of PVTT, QGY-7701 and MHCC97H cells. The invasive cells were quantified and analyzed. The scale bars were indicated. *, *P*<0.05; **, *P*<0.01; ***, *P*<0.001; ****, *P*<0.0001.

To study the role of PANK1 in the progression of HCC, Flag-tagged PANK1 was overexpressed in four HCC cell lines (MHCC97H, Huh7, QGY-7701 and PVTT) (Figure 2A). Cell growth, proliferation and invasion were evaluated. The CCK8 assay showed that PANK1 overexpression inhibited the growth of PVTT and QGY-7701cells in liquid medium (Figure 2B). Furthermore, the EdU incorporation assay showed that overexpression of PANK1 inhibited the proliferation of HCC cells, suggesting that the growth disadvantage of PVTT and QGY-7701 cells with PANK1 overexpression was possibly due to decreased proliferation (Figure 2C-D). Consistent with this finding, the soft agar assay showed that PANK1 overexpression inhibited the anchorage-independent growth of PVTT, QGY-7701, MHCC97H and Huh7 HCC cells (Figure 2E-F). In addition, the Transwell assay showed that overexpression of PANK1 reduced the invasion ability of MHCC97H, QGY-7701, and PVTT HCC cells (Figure 2G-H).

**Figure 3:**
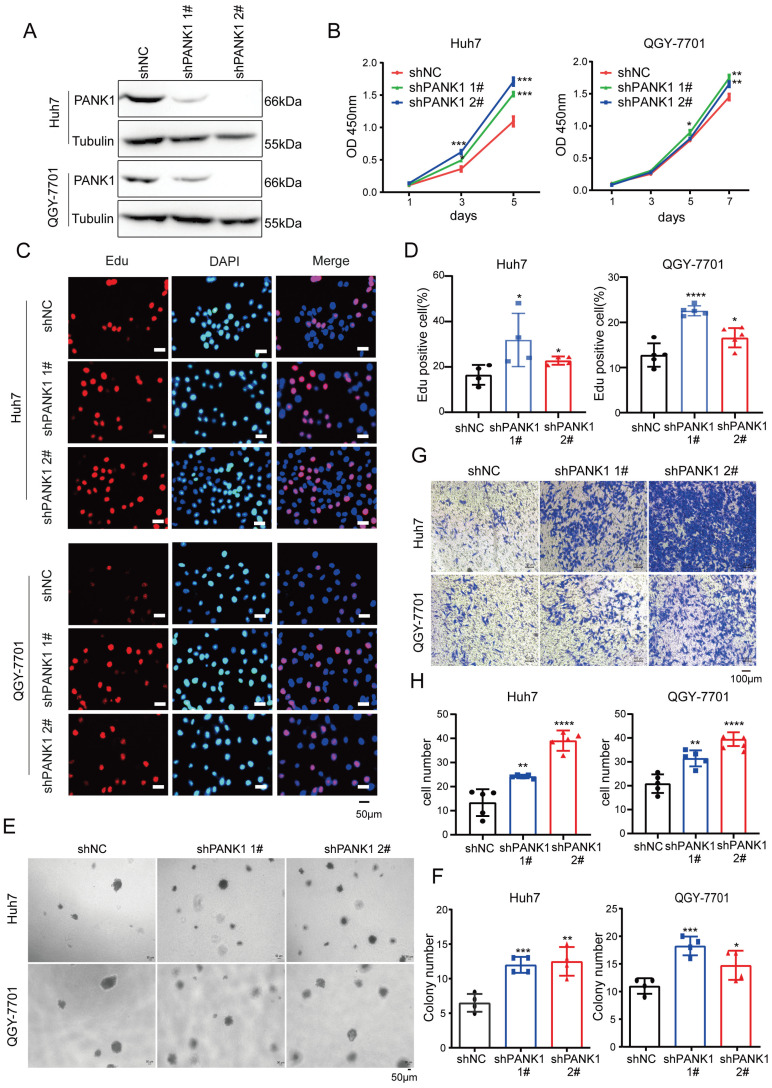
** Knocking down PANK1 promotes proliferation, growth, colony formation, and invasion of HCC cells. (A)** PANK1 expression was knocked down in Huh7 and QGY-7701 cells. The cells were infected with the shPANK1 lentivirus. After puromycin screening, expression of PANK1 in the surviving cells was measured by western blot. **(B)** A CCK8 assay was performed to measure the effect of interfering with PANK1 on growth of the HCC cells Huh7 and QGY-7701. **(C-D)** EdU assay was performed to quantify and analyze the effect of knocking down PANK1 expression on the proliferation of Huh7 and QGY-7701 cells. **(E-F)** A soft agar colony formation assay was performed to quantify and analyze the effect of knocking down PANK1 expression on the colony formation of Huh7 and QGY-7701 cells. **(G-H)** Invasion assay using the Transwell was conducted to quantify and analyze the effect of knocking down PANK1 expression on the migration ability of Huh7 and QGY-7701 cells. The scale bars were indicated. *, *P*<0.05; **, *P*<0.01; ***, *P*<0.001; ****, *P*<0.0001.

To study the role of endogenous PANK1 in HCC cells, PANK1 expression was knocked down by shRNA in Huh7 and QGY-7701 cells (Figure 3A). Consistent with the above results, knocking down the expression of PANK1 in Huh7 and QGY-7701 cells promoted the growth of these cells in liquid medium (Figure 3B) and promoted the proliferation of HCC cells (Figure 3C-D). Moreover, the soft agar assay found that knocking down the expression of PANK1 increased the anchorage-independent growth of HCC cells (Figure 3E-F). Furthermore, the Transwell assay showed that knocking down the expression of PANK1 promoted cell invasion (Figure 3G-H). In summary, these assays show that PANK1 inhibits the tumorigenesis of HCC.

### PANK1 inhibits the Wnt/β-catenin signaling pathway in HCC cells

**Figure 4:**
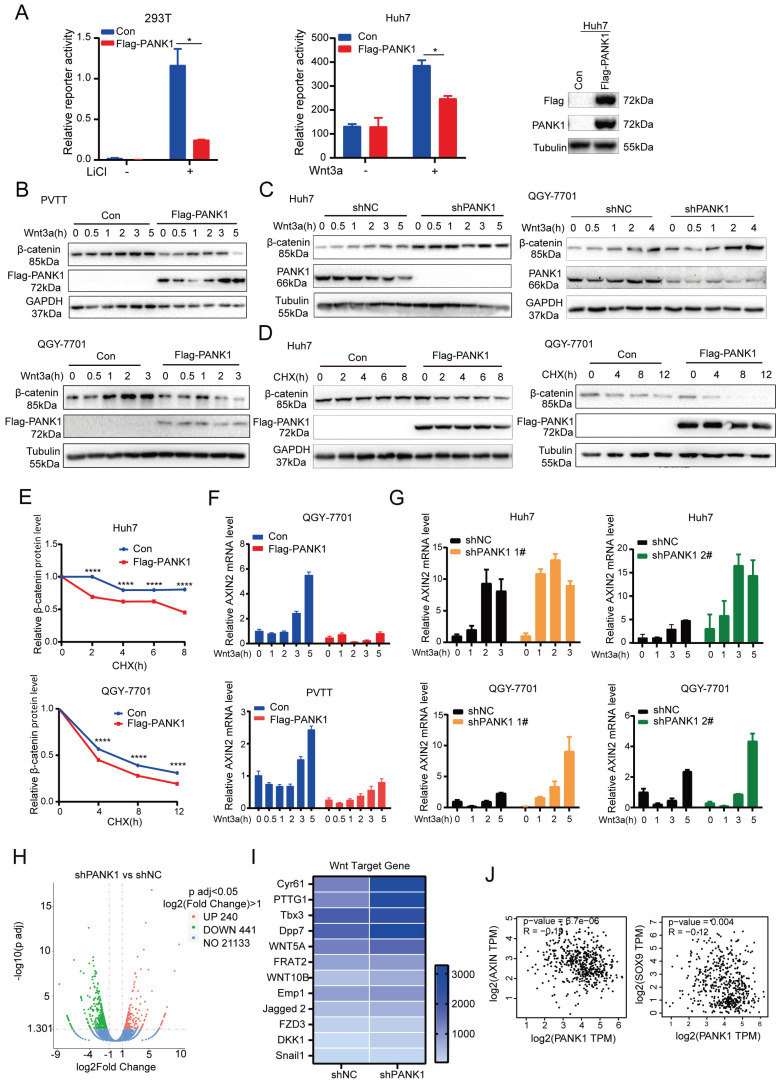
** PANK1 inhibits the Wnt/β-catenin signaling pathway. (A)** The Topflash reporter was used to detect the effect of PANK1 on the transcriptional activity of β-catenin. After transfection, the cells were stimulated by lithium chloride or Wnt3a for 8h before the measurement of the reporter activity. **(B)** The effect of overexpression of PANK1 on the level of β-catenin protein in PVTT and QGY-7701 cells was measured. After the cells were stimulated by Wnt3a for different periods of time, the β-catenin protein was measured by western blot. **(C)** The effect of knocked-down expression of PANK1 on the level of β-catenin protein in Huh7 and QGY-7701 cells was measured. After the cells were stimulated by Wnt3a for different periods of time, the β-catenin protein was measured by western blot. **(D-E)** The effect of overexpression of PANK1 in Huh7 and QGY-7701 cells on the half-life of β-catenin protein level was measured. After the cells were stimulated by CHX (50µg/ml) for different periods of time, the β-catenin protein was measured by western blot. The levels of β-catenin protein were quantified and analyze. **(F)** The effect of PANK1 overexpression in PVTT and QGY-7701 cells on the mRNA level of Axin2 was measured. After being stimulated by Wnt3a for different periods of time, the cells were collected to measure the mRNA level of Axin2. **(G)** The effect of knocked-down expression of PANK1 in Huh7 and QGY-7701 cells on the mRNA levels of Axin2 was measured. After the cells were stimulated by Wnt3a for different periods of time, they were collected to measure the mRNA level of Axin2. **(H)** RNA sequencing was performed on Huh7 cells with the knockdown of PANK1 expression, and on control cells, obtaining a volcano plot. **(I)** Differential expression of target genes of the Wnt/β-catenin signaling pathway from RNA sequencing, shown as a heat map. **(J)** As shown in the GEPIA database, PANK1 expression is negatively correlated with expression of Axin and SOX9, the target genes of Wnt/β-catenin signaling pathway. ****, *P*<0.0001.

Since it was during kinase library screening that PANK1 was discovered to be a potential kinase effective in inhibiting the Wnt/β-catenin signaling pathway, the regulation of PANK1 on the Wnt/β-catenin signaling pathway was investigated. Overexpression of PANK1 in 293T cells and Huh7 cells significantly inhibited the activation of the Topflash reporter, suggesting that PANK1 inhibits the transcriptional activity of β-catenin (Figure 4A). Further study revealed that overexpression of PANK1 inhibited Wnt3a-induced accumulation of β-catenin and that knockdown of PANK1 expression enhanced Wnt3a-induced accumulation of β-catenin (Figure 4B-C). To further study the effect of PANK1 expression on the stability of β-catenin, cells were treated with CHX to determine the effect of PANK1 expression on the half-life of β-catenin. The results showed that overexpression of PANK1 reduced the half-life of β-catenin (Figure 4D-E). These findings indicate that PANK1 inhibits the Wnt/β-catenin signaling pathway by regulating the stability of β-catenin.

Axin2 is a target gene of the Wnt/β-catenin signaling pathway. PANK1 overexpression reduced the mRNA level of Axin2, while the mRNA level of Axin2 was increased by knocking down the expression of PANK1 (Figure 4F-G). Next, RNA sequencing was performed on control cells and Huh7 cells with knockdown of PANK1, revealing differences in the expression of 681 genes in Huh7 cells with knockdown of PANK1, among which 240 genes were upregulated (Figure 4H). Some of these upregulated genes were target genes of the Wnt/β-catenin signaling pathway, for example, Cyr61, PTTG1, Tbx3, WNT5A, FRAT2, and Emp1 (Figure 4I). These genes are widely involved in cell growth and motility. In the GEPIA database, it was also observed that the mRNA level of PANK1 was negatively correlated with those of SOX9 and Axin (Figure 4J). These findings indicate that PANK1 reduces the stability of β-catenin, thereby inhibiting its transcriptional activity.

### PANK1 interacts with the β-catenin destruction complex and phosphorylates the N-terminal serine/threonine residues of β-catenin

**Figure 5:**
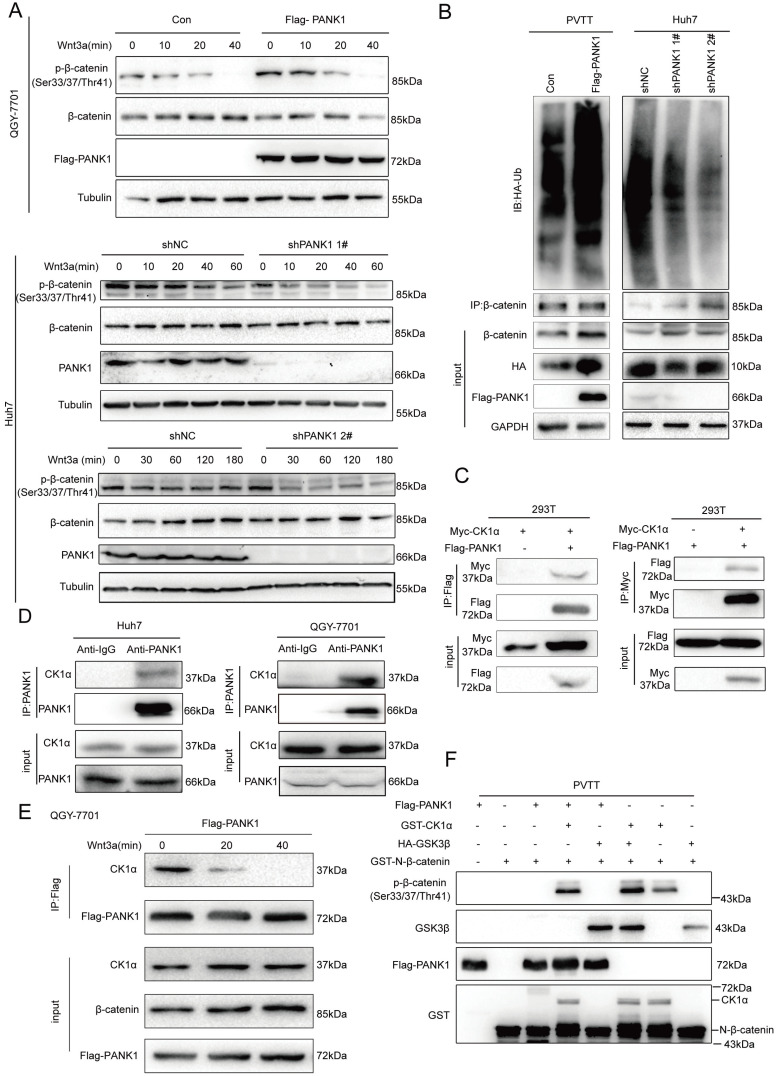
** PANK1 phosphorylates the N-terminal serine and threonine residues of β-catenin. (A)** The effect of knockdown or overexpression of PANK1 in Huh7 and QGY-7701 cells on the phosphorylation level of β-catenin was measured. After the cells were stimulated by Wnt3a for different periods of time, the phosphorylation level of β-catenin was measured by western blot. **(B)** Ubiquitin assay was performed to measure the effect of PANK1 expression on the ubiquitination level of β-catenin. After being treated with MG132 for 10h, the PVTT cells with overexpressed PANK1, Huh7 cells, with down-regulated expression of PANK1, and their respective control cells were lysed for supernatant collection. The antibody against β-catenin was used for CO-IP. Then, a western blot assay was performed, and the antibody against ubiquitin was used for measurement. **(C)** The interaction between exogenously expressed CK1α (Myc-CK1α) and PANK1 (Flag-PANK1) was measured in 293T cells. The Myc-CK1α and Flag-PANK1 plasmids were transiently transfected into 293T cells. 48h later, CO-IP was performed with an antibody against Myc or the Flag tag. **(D)** The interaction between endogenous CK1α and PANK1 in Huh7 and QGY-7701 cells was measured. **(E)** The Wnt3a stimulation-dependent interaction between CK1α and PANK1 in QGY-7701 cells was measured. After treatment with Wnt3a for different periods of time, the QGY-7701 cells with the overexpression PANK1 were lysed and centrifuged for supernatant collection. The antibody against Flag was used for CO-IP. **(F)** The phosphorylation of β-catenin by PANK was measured through the in vitro kinase reaction. See “Materials and Methods” for the details on the reaction.

As discovered above, PANK1 affects the stability of β-catenin. Then, whether PANK1 expression affected the degradation of β-catenin via the ubiquitination pathway was explored. Phosphorylation of the N-terminal serine/threonine residues of β-catenin is essential for its ubiquitination 24. Therefore, the effect of PANK1 expression on the phosphorylation of the N-terminal serine/threonine residues of β-catenin was valuated. Overexpression of PANK1 in QGY-7701 cells promoted the phosphorylation of the N-terminal serine/threonine residues of β-catenin; while knocking down PANK1 expression in Huh7 cells inhibited this phosphorylation (Figure 5A). Consistently, PANK1 overexpression increased the ubiquitination level of β-catenin, while the ubiquitination level of β-catenin was reduced by PANK1 inhibition (Figure 5B). These results indicate that PANK1 promotes β-catenin degradation by promoting the phosphorylation of the N-terminal serine/threonine residues of β-catenin.

Phosphorylation of the serine/threonine residues at the N-terminus of β-catenin occurs in the β-catenin destruction complex. Next, we examined the interactions between PANK1 and the components of the β-catenin destruction complex. Exogenous Flag-PANK1 overexpressed in 293T cells interacted with Myc-CK1α (Figure 5C). However, no interaction between PANK1 and other components in the Wnt/β-catenin pathway (RNF43, AXIN1, LRP6, β-catenin, DVL2 and GSK3β) was observed (Figure S3A-F). The interaction between endogenously expressed PANK1 and CK1α was also detectable in QGY-7701 and Huh7 cells (Figure 5D). Moreover, the interaction between PANK1 and CK1α was regulated by Wnt3a stimulation. Wnt3a stimulation weakened the interaction between these proteins (Figure 5E). Considering that PANK1 is a kinase and interacts with the components of the β-catenin destruction complex, we further confirmed whether PANK1 directly phosphorylates the serine/threonine residues in the N-terminus of β-catenin. The in vitro kinase assay results showed that PANK1 did not phosphorylate the residues alone, but instead cooperated with CK1α to phosphorylate them (Figure 5F). The synergistic effect was equivalent to that of GSK3β and CK1α. This indicates that PANK1 promotes β-catenin degradation by phosphorylating the N-terminal serine/threonine residues of β-catenin, thereby inhibiting the Wnt/β-catenin signaling pathway.

### PANK1 inhibits the tumorigenicity of HCC via Wnt/β-catenin signaling pathway

**Figure 6:**
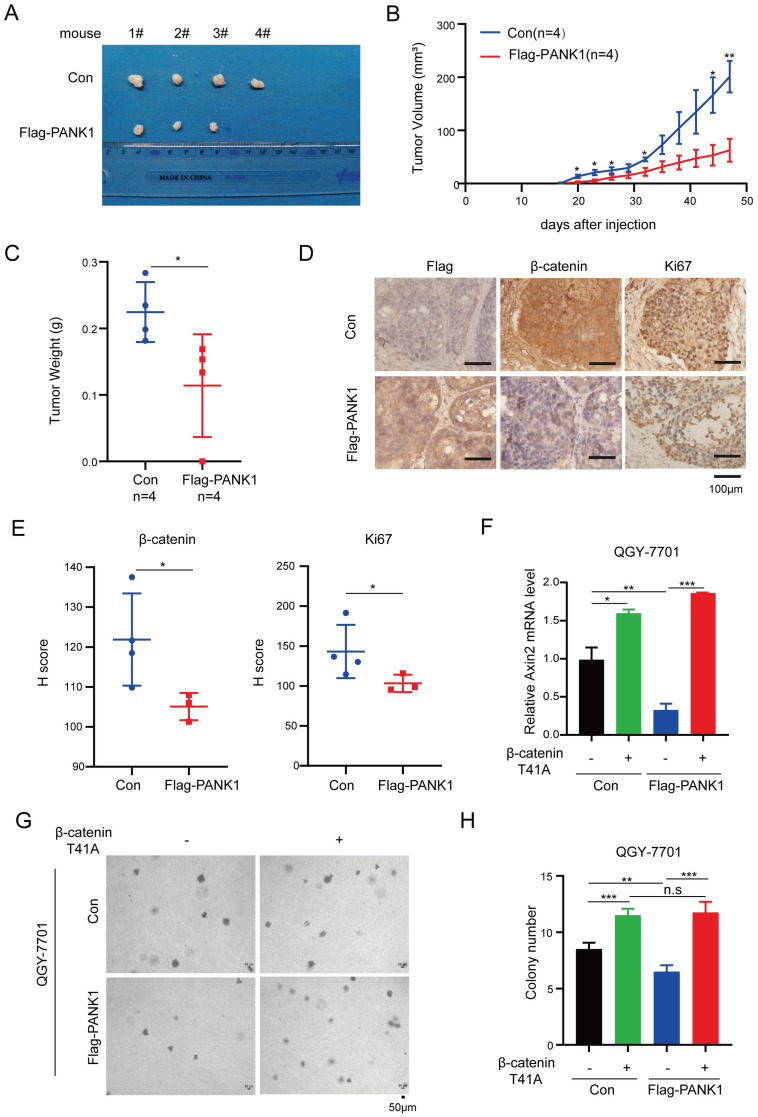
** PANK1 impairs the tumorigenicity of HCC cells. (A)** The morphology of the xenografts formed by the Huh7 cells with the overexpression of PANK1 or the control cells. See materials and methods for details. **(B)** Growth curve of xenograft tumors. Tumor volumes were measured at indicate time points. (n=4, Error bars represent SEM, *P<0.05, paired t-test). **(C)** The weight of the xenografts was examined. * P<0.05, paired t-test. **(D-E)** Representative images of immunohistochemical (IHC) staining for Flag-PANK1, β-catenin and Ki67 in xenografts. Scale bar,100 µm. The H-Scores of (D) were analyzed. **(F)** qPCR assay was performed to measure the rescue effect of β-catenin (T41A) on the mRNA level of Axin2 in QGY-7701 cells with overexpressed PANK1. **(G-H)** A soft agar assay was performed to measure the rescue effect of β-catenin (T41A) on colony formation of QGY-7701 cells with overexpressed PANK1. Colonies were counted and the analyzed. The scale bars were indicated. *, *P*<0.05; **, *P*<0.01; ***, *P*<0.001.

To explore the functions of PANK1 in the tumorigenicity of HCC cells, the tumorigenicity of Huh7 cells with the overexpression of PANK1 and control cells was examined in the nude mice. Overexpression of PANK1 significantly impaired the tumorigenicity of Huh7 cells, which was demonstrated by size, growth curve and weight of the xenograft (Figure 6A-C). Moreover, decreased levels of β-catenin and Ki67 proteins were observed in the tumors (Figure 6D-E), indicating PANK1 inhibited the tumorigenicity of HCC through negatively regulating Wnt/β-catenin signaling pathway.

To further investigate whether PANK1 suppresses the malignant phenotype of HCC cells by inhibiting Wnt/β-catenin signaling, constitutively active β-catenin (T41A) was overexpressed in cells with overexpression of PANK1. As shown in Figure 6F, overexpression of constitutively active β-catenin (T41A) overcame the inhibitory effect of PANK1 on the expression of Axin2 (Figure 6F). Moreover, overexpression of constitutively active β-catenin (T41A) overcame the inhibitory effects of PANK1 on the anchorage-independent growth (Figure 6G-H). This indicates that PANK1 inhibits the growth and colony formation of HCC cells by negatively regulating Wnt/β-catenin signaling.

### The expression of β-catenin and PANK1 predicts the prognosis of HCC patients

**Figure 7:**
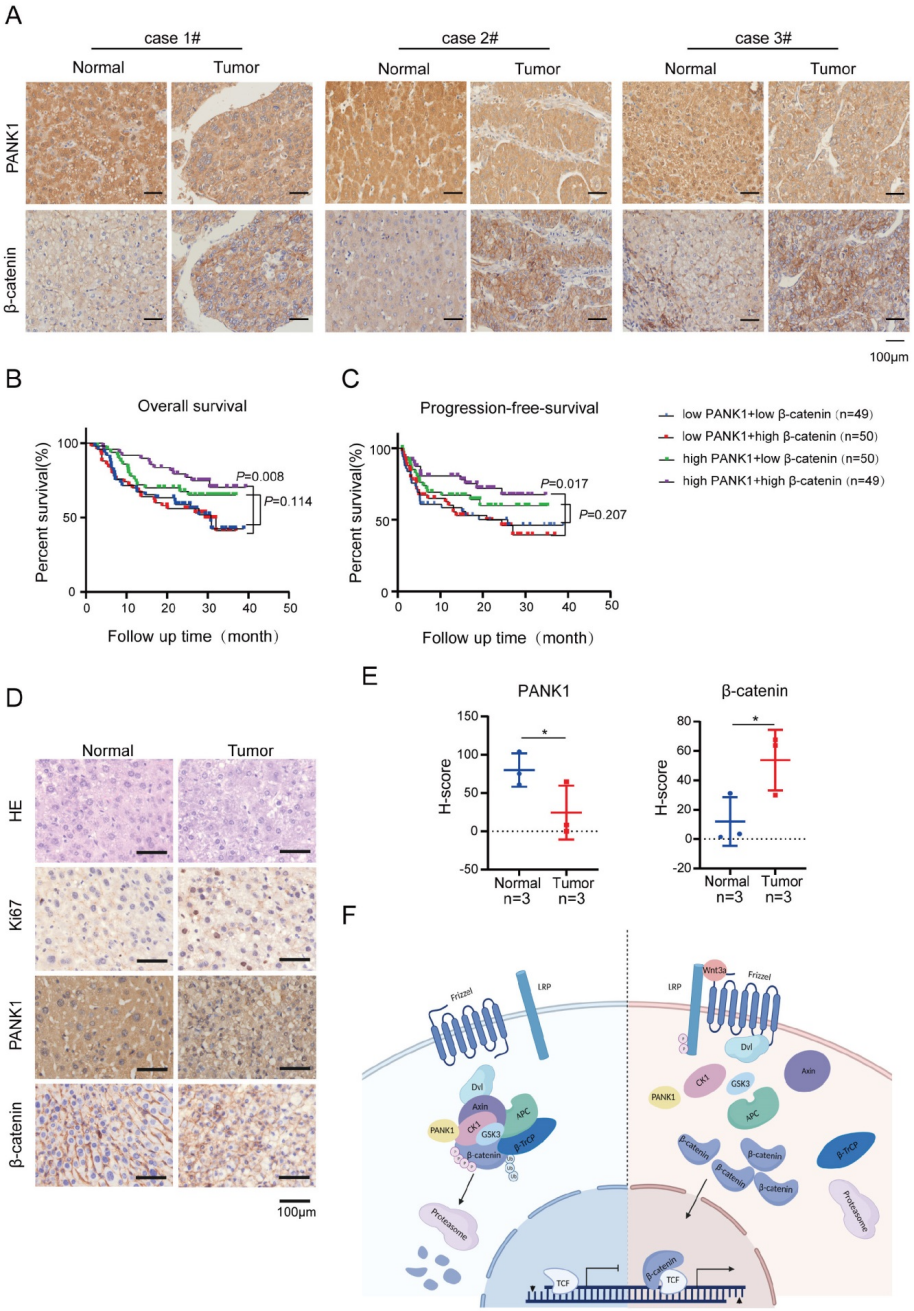
** The expression of PANK1 is negatively correlated with the level of β-catenin protein in HCC. (A)** IHC was used to measure the level of PANK1 and β-catenin proteins in 3 cases of HCC tissues (tumor) and adjacent tissues (normal). **(B-C)** Overall survival analysis and progression-free survival analysis. The expression of PANK1 and β-catenin in the HCC tissue arrays was measured by IHC and scored. The patients were divided into 4 groups according to their PANK1 and β-catenin scores to analyze the differences in overall survival and progression-free survival among each group. **(D-E)** IHC was used to measure the levels of PANK1, β-catenin proteins, staining cell proliferation marker Ki67 in DEN-induced HCC model mice. The expression of PANK1 and β-catenin was scored and analyzed. **(F)** Model showing how PANK1 inhibits the Wnt/β-catenin signaling pathway. It interacts with CK1α, phosphorylating the N-terminal serine and threonine residues of β-catenin, thereby inhibiting the pathway. The scale bars were indicated. *, *P*<0.05.

To further explore the correlation between the expression of PANK1 and β-catenin, the protein levels of PANK1 and β-catenin in the HCC tissue array were examined. In the HCC tissue array, the level of β-catenin protein was increased in the HCC tissues, as PANK1 expression was downregulated in the HCC tissues (Figure 7A).

Subsequently, the effect of PANK1 and β-catenin expression on the prognosis of HCC patients was analyzed. The results showed that among patients with high expression of β-catenin, the overall survival times of those with high expression of PANK1 were significantly longer than those of patients with low PANK1 expression (Figure 7B). Furthermore, analysis of progression-free survival showed that among patients with high expression of β-catenin, the progression-free survival times of patients with high expression of PANK1 were significantly longer than those of patients with low PANK1 expression (Figure 7C). Additionally, in the mouse model of DEN-induced HCC, PANK1 expression was observed to be significantly downregulated in HCC tissues (Figure 7D-E), accompanying by the increase of cytoplasmic and/or nuclear β-catenin. Taken together, these data further support the role of PANK1 in HCC and its negative regulatory effect on the Wnt/β-catenin pathway.

## Discussion

PANK1 is the rate-limiting enzyme in the synthesis of CoA 21. By phosphorylating pantothenic acid, it regulates de novo synthesis of CoA and the ratio of acetyl-CoA to CoA in cells, thereby exerting an important influence on the acetylation modification of proteins and fatty acid metabolism 21. PANK1 is the target gene of P53 20. Knockout of PANK1 leads to impairment of fatty acid β-oxidation and gluconeogenesis in mice 18. The role of PANK1 in tumors has rarely been reported. As observed in the present study, PANK1 expression is downregulated in clinical HCC specimens, and its expression level is significantly correlated with prognosis. PANK1 inhibits the growth, invasion, and tumorigenic ability of HCC cells. As revealed in this study, PANK1 phosphorylates the N-terminal serine and threonine residues of β-catenin, promoting degradation of β-catenin through the ubiquitination pathway (Figure 7F). These findings reveal the effect of PANK1 on tumor progression, increasing the understanding of its biological functions.

The serine/threonine kinase activity of PANK1 is one of the important findings of this study. Existing reports show that PANK1 uses pantothenic acid as substrate, and catalyzes the rate-limiting reaction in de novo synthesis of CoA 20. The present study found that PANK1 can synergize with CK1α to promote the phosphorylation of β-catenin, revealing a new substrate of PANK1. It is often reported that metabolic kinases function as protein kinase. Under pathological conditions (including tumors), some metabolic enzymes have been reported to have nonclassical or even metabolism-irrelevant biological functions (moonlighting functions) 25, 26. Through these functions, these enzymes participate in DNA repair, cell proliferation, and remodeling of the tumor microenvironment, thus promoting disease progression.

For example, PGAM1 interacts with the phospholipase WIP1 and therefore retains it in the cytoplasm to prevent it from dephosphorylating ATM, thus promoting DNA repair 25; in addition, PKM2 directly phosphorylates Ser95 of SNAP23 to promote the release of exosomes, thus remodeling the tumor microenvironment 26. Recently, it was reported that the phosphoenolpyruvate carboxylase PCK1 directly phosphorylates Ser207 of Insig1 and Ser151 of Insig2 to promote lipid metabolism 27. These findings indicate that the moonlighting functions of metabolic enzymes play very important roles in promoting disease progression under pathological conditions. The regulation of Wnt/β-catenin signaling pathway by metabolic kinases has already been reported. EGF stimulation induces PKM2 to enter the nucleus, where it interacts with β-catenin to promote the transcription of CCND1 and other downstream target genes of β-catenin 28. The present study reveals that PANK1 inhibits the progression of HCC via its protein kinase activity.

In the present study, the in vitro kinase assay showed that PANK1 and CK1α cooperate to phosphorylate the N-terminal serine/threonine residues of β-catenin. This effect is similar to the synergistic effect of GSK3β and CK1α 29. In the Wnt/β-catenin signaling pathway, GSK3β can localize in the β-catenin destruction complex to phosphorylate β-catenin and inhibit the Wnt/β-catenin signaling pathway; it can also localize near the cell membrane to phosphorylate LRP6 and activate the Wnt/β-catenin signaling pathway. When the Wnt ligand binds to the LRP6 receptor, GSK3β is rapidly recruited into the signalosome near the cell membrane 9, 30. Owing to the presence of PANK1, a buffering process exists for Wnt3a-induced activation of β-catenin. Consistent with this observation, in the coimmunoprecipitation (co-IP) assay, the interaction between CK1α and PANK1 was observed to slowly weaken with Wnt3a stimulation. From this perspective, PANK1 is likely to have a “braking effect” on Wnt/β-catenin signaling pathway activation.

PANK1 activity is inhibited by metabolites such as CoASH, acetyl-CoA, malonyl-CoA and long-chain acetyl-CoA 22. Many studies have reported that the levels of these metabolites are increased in obese people 31, 32. Obesity has been found to be a risk factor for HCC 33, 34. These metabolites are likely to inhibit the activity of PANK1, thus activating the Wnt/β-catenin signaling pathway and promoting the occurrence and progression of HCC. Thus, the findings of the present study explain why obese people have a higher risk of HCC.

In conclusion, the present study reveals that PANK1 expression is downregulated in HCC, which promotes the progression of HCC by activating the Wnt/β-catenin signaling pathway. This finding suggests that HCC may also be treated by enzymatically activating PANK1.

## Supplementary Material

Supplementary figures and tables.

## Figures and Tables

**Table 1:** The correlation between PANK1 expression and the clinical features of HCC.

Characteristic	Total	PANK1 Expression		χ^2^	*P*
		Down-regulated (n=100)	Up-regulated (n=99)		
**Gender**				0.167	0.432
Male	184	91	93		
Female	15	9	6		
**Age (years)**				0.042	0.838
≤50	114	58	56		
>50	85	42	43		
**BMI (kg/m** ^2^ **)**				0.13	0.937
≤18.5	9	5	4		
18-25	127	64	63		
≥25	63	31	32		
**Tumor size**				4.756	0.029*
≤11	170	80	90		
>11	29	20	9		
**Tumor number**				0.447	0.504
≤1	174	89	85		
≥2	25	11	14		
**Paracancerous microtumor**				0.472	0.492
No	128	62	66		
Yes	71	38	33		
**Intratumoral hemangioma**				0.007	0.933
No	116	58	58		
Yes	83	42	41		
**Ascites**				2.133	0.144
No	181	88	93		
Yes	18	12	6		
**Cirrhosis**				0.439	0.565
Ⅰ-Ⅱ	118	57	61		
Ⅲ-Ⅳ	81	43	38		
**PVTT**				0.447	0.529
No	174	89	85		
Yes	25	11	14		
**Capsular invasion**				0.533	0.465
Ⅰ-Ⅱ	140	68	72		
Ⅲ	59	32	27		
**Tumor grade**				1.044	0.307
Ⅰ-Ⅱ	50	22	28		
Ⅲ	149	78	71		

*, *P*<0.05

## References

[1] Siegel RL, Miller KD, Fuchs HE, Jemal A (2021). Cancer Statistics, 2021. CA: a cancer journal for clinicians.

[2] Bianco C, Casirati E, Malvestiti F, Valenti L (2021). Genetic predisposition similarities between NASH and ASH: Identification of new therapeutic targets. JHEP reports: innovation in hepatology.

[3] Luu HN, Neelakantan N, Geng TT, Wang R, Goh GB, Clemente JC (2021). Quality diet indexes and risk of hepatocellular carcinoma: Findings from the Singapore Chinese Health Study. International journal of cancer.

[4] Masuzaki R, Karp SJ, Omata M (2016). NAFLD as a risk factor for HCC: new rules of engagement?. Hepatology international.

[5] Donato F, Gelatti U, Chiesa R, Albertini A, Bucella E, Boffetta P (1999). A case-control study on family history of liver cancer as a risk factor for hepatocellular carcinoma in North Italy. Brescia HCC Study. Cancer causes & control: CCC.

[6] Tagger A, Donato F, Ribero ML, Chiesa R, Portera G, Gelatti U (1999). Case-control study on hepatitis C virus (HCV) as a risk factor for hepatocellular carcinoma: the role of HCV genotypes and the synergism with hepatitis B virus and alcohol. Brescia HCC Study. International journal of cancer.

[7] Liu ZZ, Yan LN, Dong CN, Ma N, Yuan MN, Zhou J (2019). Cytochrome P450 family members are associated with fast-growing hepatocellular carcinoma and patient survival: An integrated analysis of gene expression profiles. Saudi journal of gastroenterology: official journal of the Saudi Gastroenterology Association.

[8] Perugorria MJ, Olaizola P, Labiano I, Esparza-Baquer A, Marzioni M, Marin JJG (2019). Wnt-beta-catenin signalling in liver development, health and disease. Nature reviews Gastroenterology & hepatology.

[9] Moon RT, Kohn AD, De Ferrari GV, Kaykas A (2004). WNT and beta-catenin signalling: diseases and therapies. Nature reviews Genetics.

[10] Allison SJ (2015). Development: beta-catenin network controls nephron patterning. Nature reviews Nephrology.

[11] Yuan K, Xie K, Lan T, Xu L, Chen X, Li X (2020). TXNDC12 promotes EMT and metastasis of hepatocellular carcinoma cells via activation of beta-catenin. Cell death and differentiation.

[12] Chen B, Lan J, Xiao Y, Liu P, Guo D, Gu Y (2021). Long noncoding RNA TP53TG1 suppresses the growth and metastasis of hepatocellular carcinoma by regulating the PRDX4/beta-catenin pathway. Cancer letters.

[13] Adebayo Michael AO, Ko S, Tao J, Moghe A, Yang H, Xu M (2019). Inhibiting Glutamine-Dependent mTORC1 Activation Ameliorates Liver Cancers Driven by beta-Catenin Mutations. Cell metabolism.

[14] Gao Q, Zhu H, Dong L, Shi W, Chen R, Song Z (2019). Integrated Proteogenomic Characterization of HBV-Related Hepatocellular Carcinoma. Cell.

[15] Bengochea A, de Souza MM, Lefrancois L, Le Roux E, Galy O, Chemin I (2008). Common dysregulation of Wnt/Frizzled receptor elements in human hepatocellular carcinoma. British journal of cancer.

[16] Ou D, Chen L, He J, Rong Z, Gao J, Li Z (2020). CDK11 negatively regulates Wnt/beta-catenin signaling in the endosomal compartment by affecting microtubule stability. Cancer biology & medicine.

[17] Liu Y, Cheng Z, Li Q, Pang Y, Cui L, Qian T (2019). Prognostic significance of the PANK family expression in acute myeloid leukemia. Annals of translational medicine.

[18] Leonardi R, Rock CO, Jackowski S (2014). Pank1 deletion in leptin-deficient mice reduces hyperglycaemia and hyperinsulinaemia and modifies global metabolism without affecting insulin resistance. Diabetologia.

[19] Subramanian C, Yao J, Frank MW, Rock CO, Jackowski S (2020). A pantothenate kinase-deficient mouse model reveals a gene expression program associated with brain coenzyme a reduction. Biochimica et biophysica acta Molecular basis of disease.

[20] Wang SJ, Yu G, Jiang L, Li T, Lin Q, Tang Y (2013). p53-Dependent regulation of metabolic function through transcriptional activation of pantothenate kinase-1 gene. Cell cycle.

[21] Ramaswamy G, Karim MA, Murti KG, Jackowski S (2004). PPARalpha controls the intracellular coenzyme A concentration via regulation of PANK1alpha gene expression. Journal of lipid research.

[22] Rock CO, Karim MA, Zhang YM, Jackowski S (2002). The murine pantothenate kinase (Pank1) gene encodes two differentially regulated pantothenate kinase isozymes. Gene.

[23] Cai Z, Qian ZY, Jiang H, Ma N, Li Z, Liu LY (2018). hPCL3s Promotes Hepatocellular Carcinoma Metastasis by Activating beta-Catenin Signaling. Cancer research.

[24] Xue J, Chen Y, Wu Y, Wang Z, Zhou A, Zhang S (2015). Tumour suppressor TRIM33 targets nuclear beta-catenin degradation. Nature communications.

[25] Ohba S, Johannessen TA, Chatla K, Yang X, Pieper RO, Mukherjee J (2020). Phosphoglycerate Mutase 1 Activates DNA Damage Repair via Regulation of WIP1 Activity. Cell reports.

[26] Wei Y, Wang D, Jin F, Bian Z, Li L, Liang H (2017). Pyruvate kinase type M2 promotes tumour cell exosome release via phosphorylating synaptosome-associated protein 23. Nature communications.

[27] Xu D, Wang Z, Xia Y, Shao F, Xia W, Wei Y (2020). The gluconeogenic enzyme PCK1 phosphorylates INSIG1/2 for lipogenesis. Nature.

[28] Yang W, Xia Y, Ji H, Zheng Y, Liang J, Huang W (2011). Nuclear PKM2 regulates beta-catenin transactivation upon EGFR activation. Nature.

[29] Liu C, Li Y, Semenov M, Han C, Baeg GH, Tan Y (2002). Control of beta-catenin phosphorylation/degradation by a dual-kinase mechanism. Cell.

[30] Schunk SJ, Floege J, Fliser D, Speer T (2021). WNT-beta-catenin signalling - a versatile player in kidney injury and repair. Nature reviews Nephrology.

[31] Chen Y, Chen J, Zhang C, Yang S, Zhang X, Liu Y (2019). Deficiency in the short-chain acyl-CoA dehydrogenase protects mice against diet-induced obesity and insulin resistance. FASEB journal: official publication of the Federation of American Societies for Experimental Biology.

[32] Softic S, Meyer JG, Wang GX, Gupta MK, Batista TM, Lauritzen H (2019). Dietary Sugars Alter Hepatic Fatty Acid Oxidation via Transcriptional and Post-translational Modifications of Mitochondrial Proteins. Cell metabolism.

[33] Ioannou GN (2021). Epidemiology and risk-stratification of NAFLD-associated HCC. Journal of hepatology.

[34] Brahma MK, Gilglioni EH, Zhou L, Trepo E, Chen P, Gurzov EN (2021). Oxidative stress in obesity-associated hepatocellular carcinoma: sources, signaling and therapeutic challenges. Oncogene.

