# Supplementary material for: Pantothenate Kinase 1 Inhibits the Progression of Hepatocellular Carcinoma by Negatively Regulating Wnt/β-catenin Signaling
Source: Int J Biol Sci. 2022 Jan 24;18(4):1539–54. doi: 10.7150/ijbs.67842 (PMC8898371; doi:10.7150/ijbs.67842)
Supplement: Supplementary file 1 — Supplementary figures and tables. [file ijbsv18p1539s1.pdf]

Supplementary materials inventory

The supplementary materials included 3 supplementary Tables and 3 supplementary Figures.

Supplementary Table

Table S1: Summary of Clinicopathologic Variables of 72 HCC patients.

Table S2: The primer sequences for clone and qPCR.

Table S3: Summary of Clinicopathologic Variables of 199 HCC patients in the tissue array.

Supplementary Figure

Figure S1 Down-regulation of PANK1 in the HCC. (A) The screening strategy for PANK1. (B) The expression of PANK1 between normal and primary HCC tumor in TCGA database. (C) Survival analysis of PANK1 in KM Plotter database. (D) The expression of PANK1 in normal tissues in The Human Protein Atlas database.

Figure S2 The correlations between the expression of PANK1 and serum bio-marker of 199 HCC patients in the tissue array. The contents of Alpha fetal protein (AFP) (A), α-L-focusidase (AFU) (B), Alkaline phosphatase (ALP) (C), Alanine aminotransferase (ALT) (D), Aspartate aminotransferase (AST) (E), Carbohydrate antigen199 (CA199) (F), Carcino-embryonic antigen (CEA) (G), Gamma-glutamyl transpeptidase (GGT) (H), Hepatitis B virus (HBV) DNA (I), Albumin (J),

Prothrombin time (PT) (K), Direct Bilirubin (DBIL) (L), and Total bilirubin (TBIL) (M) were measured and the correlations were analyzed.

Figure S3 The interaction between exogenously expressed PANK1 (Flag-PANK1) and RNF43 (A), AXIN1 (B), LRP6 (C), β-catenin (D), DVL2 (E), GSK3β (F) was examined in 293T cells.

Supplementary Table S1 Summary of Clinicopathologic Variables of 72 HCC patients

Characteristic	No. of Patients
**Gender**	
Male	60
Female	12
**Age (years)**	
<55	26
≥55	46
**Tumor size (cm)**	
<55	31
≥5	41
**Tumor number**	
≤1	60
≥2	12
**HBV infection**	
Yes	62
No	10
**Organ metastasis**	
Yes	7
No	65
**Edmondson grading**	
I/II	2
II/III	40
III/IV	30
**Microvascular invasion (MVI)**	
M0	19
M1	35
M2	18

Supplementary Table S2 The primer sequences for clone and qPCR

Gene (homo)	Forward (5’-3’)	Reverse (5’-3’)
**PANK1 (clone)**	GGATCCATGTTGAAACTCGTC	GAATTCCTACTTGTCATCAGT
**shPANK1 1# (clone)**	CCGGGCCTGCTTTATGTCGACTCTGCTCGAGCAGAGTCGACATAAAGCAGGCTTTTTG	AATTCAAAAAGCCTGCTTTATGTCGACTCTGCTCGAGCAGAGTCGACATAAAGCAGGC
**shPANK1 2# (clone)**	CCGGGCTATGCACAGGTCATTCAGCTCGAGCTGAATGAACCTGTGCATAGCTTTTTG	AATTCAAAAAGCTATGCACAGGTTCATTCAGCTCGAGCTGAATGAACCTGTGCATAGC
**PANK1 (qPCR)**	CTGCCTTGATAACCCATACCCT	CTTGGAGTACACGGCTAGAATG
**GAPDH (qPCR)**	TGTTGCCATCAATGACCCCTT	CTCCACGACGTACTCAGCG
**Axin2 (qPCR)**	TACACTCCTTATTGGGCGATCA	TTGGCTACTCGTAAAGTTTTGGT
**18S (qPCR)**	AGGCCCTGTAATTGGAATGAGTC	GCTCCCAAGATCCAACTACGAG

Supplementary Table S3 Summary of Clinicopathologic Variables of 199 HCC patients in the tissue array.

Characteristic	No. of Patients
**Gender**	
male	184
female	15
**Age (years)**	
≤50	114
>50	85
**BMI (kg/m^2)**	
≤18.5	9
18.5-25	127
≥25	63
**Tumor size (cm)**	
≤11	170
>11	29
**Tumor number**	
≤1	174
≥2	25
**Paracancerous microtumor**	
No	128
Yes	71
**Intracancerous hemangioma**	
No	116
Yes	83
**Ascites**	
No	181
Yes	18
**Cirrhosis**	
I-II	118
III-IV	81
**PVTT**	
No	174
Yes	25
**Capsular invasion**	
I-II	140
III	59
**Tumor grade**	
I-II	50
III	149
**Recurrence**	
No	57
Yes	142

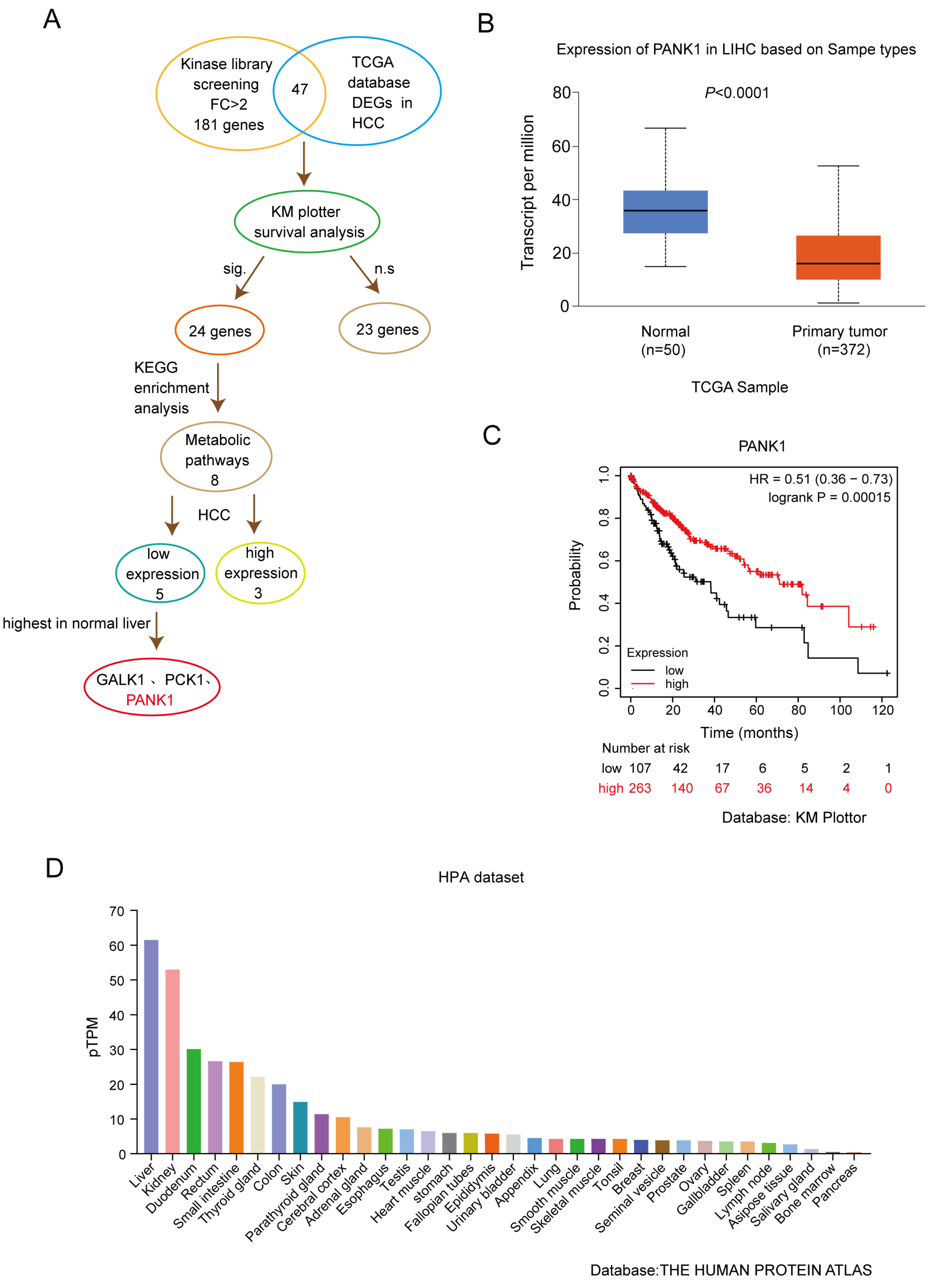
A
Kinase library screening FC>2 181 genes
47
TCGA database DEGs in HCC
KM plotter survival analysis
sig.
n.s
24 genes
23 genes
KEGG enrichment analysis
Metabolic pathways 8
HCC
low expression 5
high expression 3
highest in normal liver
GALK1、PCK1、PANK1
B
Expression of PANK1 in LIHC based on Sampe types
P<0.0001
Transcript per million
80
60
40
20
0
Normal (n=50)
Primary tumor (n=372)
TCGA Sample
C
PANK1
HR = 0.51 (0.36 − 0.73)
logrank P = 0.00015
Probability
0.0
0.2
0.4
0.6
0.8
1.0
Expression
low
high
0
20
40
60
80
100
120
Time (months)
Number at risk
low 107 42 17 6 5 2 1
high 263 140 67 36 14 4 0
Database: KM Plottor
D
HPA dataset
pTPM
70
60
50
40
30
20
10
0
Liver
Kidney
Duodenum
Rectum
Small intestine
Thyroid gland
Colon
Skin
Parathyroid gland
Cerebral cortex
Adrenal gland
Esophagus
Testis
Heart muscle
stomach
Fallopian tubes
Epididymis
Urinary bladder
Appendix
Lung
Smooth muscle
Skeletal muscle
Tonsil
Breast
Seminal vesicle
Prostate
Ovary
Gallbladder
Spleen
Lymph node
Asipose tissue
Salivary gland
Bone marrow
Pancreas
Database:THE HUMAN PROTEIN ATLAS

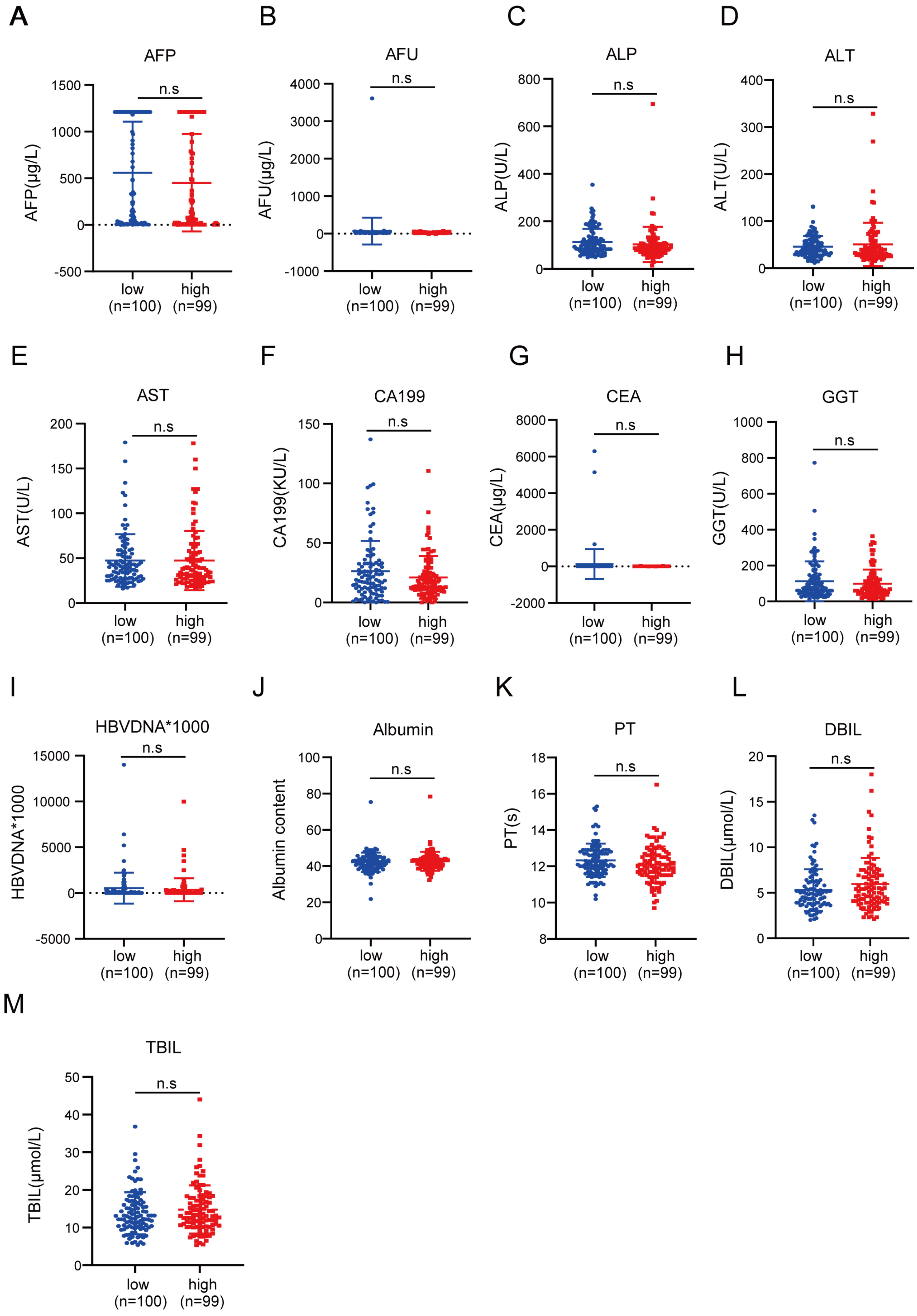
A
AFP
AFP(μg/L)
1500
1000
500
0
-500
n.s
low
(n=100)
high
(n=99)
B
AFU
AFU(μg/L)
4000
3000
2000
1000
0
-1000
n.s
low
(n=100)
high
(n=99)
C
ALP
ALP(U/L)
800
600
400
200
0
n.s
low
(n=100)
high
(n=99)
D
ALT
ALT(U/L)
400
300
200
100
0
n.s
low
(n=100)
high
(n=99)
E
AST
AST(U/L)
200
150
100
50
0
n.s
low
(n=100)
high
(n=99)
F
CA199
CA199(KU/L)
150
100
50
0
n.s
low
(n=100)
high
(n=99)
G
CEA
CEA(μg/L)
8000
6000
4000
2000
0
-2000
n.s
low
(n=100)
high
(n=99)
H
GGT
GGT(U/L)
1000
800
600
400
200
0
n.s
low
(n=100)
high
(n=99)
I
HBVDNA*1000
HBVDNA*1000
15000
10000
5000
0
-5000
n.s
low
(n=100)
high
(n=99)
J
Albumin
Albumin content
100
80
60
40
20
0
n.s
low
(n=100)
high
(n=99)
K
PT
PT(s)
18
16
14
12
10
8
n.s
low
(n=100)
high
(n=99)
L
DBIL
DBIL(μmol/L)
20
15
10
5
0
n.s
low
(n=100)
high
(n=99)
M
TBIL
TBIL(μmol/L)
50
40
30
20
10
0
n.s
low
(n=100)
high
(n=99)

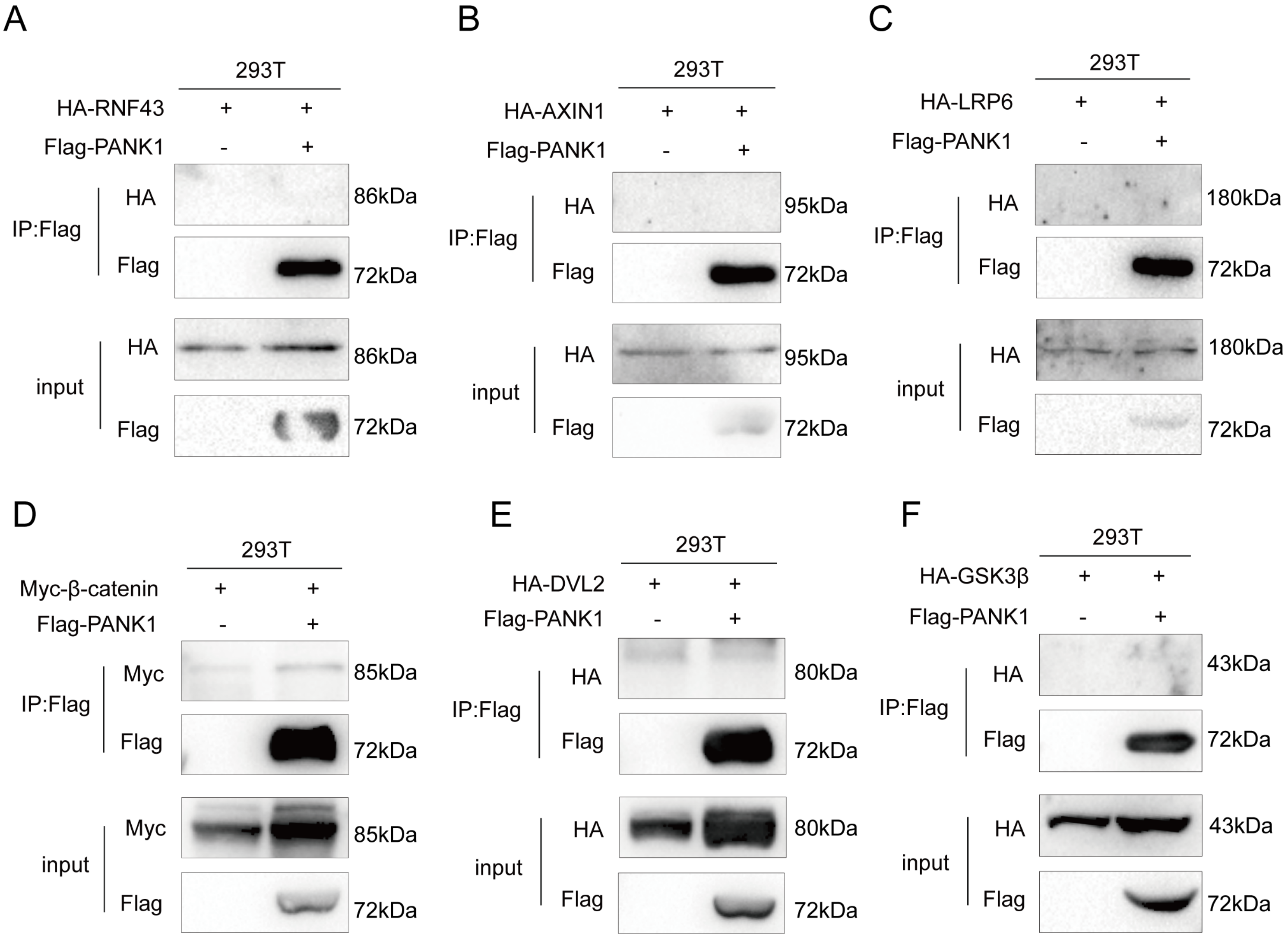
A
293T
HA-RNF43 + +
Flag-PANK1 - +
IP:Flag
HA
Flag
86kDa
72kDa
input
HA
Flag
86kDa
72kDa
B
293T
HA-AXIN1 + +
Flag-PANK1 - +
IP:Flag
HA
Flag
95kDa
72kDa
input
HA
Flag
95kDa
72kDa
C
293T
HA-LRP6 + +
Flag-PANK1 - +
IP:Flag
HA
Flag
180kDa
72kDa
input
HA
Flag
180kDa
72kDa
D
293T
Myc-β-catenin + +
Flag-PANK1 - +
IP:Flag
Myc
Flag
85kDa
72kDa
input
Myc
Flag
85kDa
72kDa
E
293T
HA-DVL2 + +
Flag-PANK1 - +
IP:Flag
HA
Flag
80kDa
72kDa
input
HA
Flag
80kDa
72kDa
F
293T
HA-GSK3β + +
Flag-PANK1 - +
IP:Flag
HA
Flag
43kDa
72kDa
input
HA
Flag
43kDa
72kDa
